# Comparative Analysis of Monopolar Electrocautery and Ultrasonic Activated Scalpel in Laparoscopic Cholecystectomy: A Comprehensive Evaluation Using Clinical, Laboratory, and Imaging Techniques

**DOI:** 10.7759/cureus.61925

**Published:** 2024-06-07

**Authors:** Kulbhushan Jain, Divakar Goyal

**Affiliations:** 1 General Surgery, HealthCity Trauma Centre & Superspeciality Hospital, Lucknow, IND; 2 Trauma and Emergency, All India Institute of Medical Sciences, Bathinda, IND

**Keywords:** acute phase reactants, monopolar cautery, ultrasonic activated scalpel, laparoscopic cholecystectomy, gallstone

## Abstract

Introduction

Laparoscopic cholecystectomy has long been the cornerstone of gallstone treatment. Both monopolar cautery and ultrasonically activated scalpel (UAS, also known as harmonic scalpel) have been employed in the dissection of the gallbladder from its fossa during laparoscopic cholecystectomy.

Material and methods

The prospective study was conducted in the Department of Surgery at Vivekananda Institute of Medical Sciences including 200 patients equally divided among the monopolar cautery and harmonic scalpel group. Patients were observed for 48 hours post-surgery, during which temperature and pain assessment were done. Acute phase reactants were measured during this period and compared with preoperative values. On the seventh day ultrasonography was done to look for the inflammatory changes.

Results

In a study involving 200 patients, the majority fell within the age bracket of 31 to 50 years, with females constituting the predominant demographic. Notably, patients who underwent surgery with a harmonic scalpel exhibited a reduced need for analgesics. Furthermore, the use of harmonic scalpels led to noteworthy alterations in acute phase reactants, including a significant decrease in the total leucocyte count (TLC) (p=0.03), neutrophils (p=0.005), and lymphocytes (p=0.02). Additionally, patients in the UAS group experienced a significantly lesser increase in erythrocyte sedimentation rate (ESR) and C-reactive protein (CRP) values (p=0.0001). Conversely, ultrasound imaging conducted on the seventh day post-surgery did not reveal any significant differences between the two groups.

Conclusion

Laparoscopic cholecystectomy performed with a harmonic scalpel is associated with a reduced tissue response and less tissue damage compared to the monopolar group.

## Introduction

Gallstone-related conditions rank among the prevalent gastrointestinal ailments necessitating hospital care. In India, the estimated prevalence of gallstones ranges from 2% to 29% [1]. Unisa et al., in 2011, investigated the occurrence of gallbladder diseases in Northern India, specifically Uttar Pradesh and Bihar. Utilizing ultrasonography, they identified a 6.20% prevalence of gallbladder diseases. Notably, symptomatic individuals (7.12%) exhibited a higher prevalence than asymptomatic individuals (2.99%) [1]. The study highlighted a significantly heightened risk of gallstone disease in females aged over 50, those with multiple pregnancies, and a familial predisposition. Similarly, males with diabetes, regular consumption of chickpeas, and exposure to unsafe water sources were found to have an elevated risk. In the Gangetic Basin of North India, the prevalence of gallstones stood at 4.15%, with females (5.59%) being more affected than males (1.99%) [1]. Laparoscopic cholecystectomy has long been the cornerstone in gallstone treatment, undergoing significant refinements from conventional four-port to single-port procedures. This evolution also gave rise to natural orifice transluminal endoscopic surgery (NOTES), utilizing natural body openings for organ extraction. Techniques like trans anal, transvaginal, trans colonic, and trans gastric access with flexible endoscopic tools are still undergoing development. Additionally, the emergence of robotic technology has brought about promising outcomes in robotic-assisted laparoscopic cholecystectomy [2-5]. For an extensive duration, both monopolar cautery and ultrasonically activated scalpel (UAS, also known as harmonic scalpel) have been employed in the dissection of the gallbladder from its fossa during laparoscopic cholecystectomy. In the literature, the inflammatory changes following laparoscopic cholecystectomy versus open surgery have been compared [6-9]. Studies have been conducted in the past comparing the two methods, and an association has been found in decreased operative time, postoperative pain, and intraoperative complications [10-12]. However, to the best of our knowledge, none have specifically assessed the impact of different dissection methods in laparoscopic cholecystectomy on postoperative inflammation. In our recent prospective observational study, we aimed to address this gap by comparing these two dissection methods.

## Materials and methods

The study was conducted in the Department of Surgery at Vivekananda Institute of Medical Sciences after getting approval from the ethical committee with a study population of around 200 including 100 patients in each category.

Patients diagnosed with cholelithiasis, presenting with either acute or chronic cholecystitis, and admitted to the surgical ward were included in the study following confirmation by ultrasonography, regardless of age, sex, or parity. Inclusion criteria adhered to standard indications for laparoscopic cholecystectomy. However, patients with empyema of the gallbladder, gallbladder perforation, or gallstone pancreatitis were excluded, as were those who did not consent.

Laparoscopic cholecystectomy was performed after pre-anesthetic evaluation, with dissection of Calot's triangle utilizing a Maryland Dissector in all cases. Cystic duct and artery ligation were conducted using clips, followed by dissection using either monopolar cautery or harmonic scalpel. Postoperatively, patients were initially monitored in the recovery area before transfer to the surgical ward. Patients from both groups were observed for 48 hours post-surgery, with ultrasound conducted on the seventh postoperative day at the time of suture removal. Pain assessment using the Visual Analog Scale was performed every two hours during this period, with analgesics administered for scores exceeding three. Parenteral analgesics were provided within the first 24 hours, followed by oral analgesics. Temperature monitoring occurred every 12 hours during the hospital stay. C-reactive protein and hemogram studies (including hemoglobin, total leukocyte count, differential leukocyte count, platelet count, and erythrocyte sedimentation rate) were conducted 48 hours post-surgery. Ultrasonography during suture removal (typically after seven days) aimed to detect postoperative inflammatory changes (changes in the echogenicity of the liver around gallbladder fossa, any edematous change or interface thickening in gallbladder fossa, any peritoneal collection, pneumobilia, haemobilia, biloma, bilehemia, seroma, any changes in common bile duct, or any other abnormality) or tissue damage in the localized area.

The results are presented in mean ± SD and percentages. The Chi-square test was used to compare the dichotomous/ categorical variables. The unpaired t-test was used to compare the discrete variables between the groups. The paired t-test was used to compare the changes in discrete variables from preoperative to postoperative. McNemar's test was used to compare the dichotomous variables from preoperative to postoperative. A p-value of <0.05 was considered significant. The statistical analysis was done using the SPSS v25 software (IBM Inc., Armonk, New York).

## Results

A total of 200 patients who met the inclusion criteria were part of the study. The demographic characteristics, including age and sex, are detailed in Table 1.

**Table 1:** Age and sex distribution between two groups

Variable	Harmonic (n=100)		Monopolar (n=100)		p-value^1^
	N	%	N	%	
Age in years					
<20	1	1	2	2	0.84
20-30	17	17	16	16	
31-40	20	20	20	20	
41-50	35	35	29	29	
51-60	18	18	19	19	
>60	9	9	14	14	
Mean±SD	44.00±12.91		45.75±14.17		
Sex					
Male	29	29	24	24	0.42
Female	71	71	76	76	

These patients were admitted to the surgical ward during the postoperative period, where their pain levels and temperatures were monitored. Temperature variations and analgesic requirements (both oral and intravenous) are presented in Table 2.

**Table 2:** Comparison of temperature changes and amount of analgesic requirement in ultrasonic activated scalpel (harmonic) and monopolar groups

Variables	Harmonic (n=100)		Monopolar (n=100)		p-value^1^
	N	%	N	%	
12 hour					
Normal	97	97	98	98	0.84
Increased	3	3	2	2	
24 hour					
Normal	100	100	100	100	NA
Increased	0	0	0	0	
36 hour					
Normal	100	100	100	100	NA
Increased	0	0	0	0	
48 hour					
Normal	100	100	100	100	NA
Increased	0	0	0	0	
Analgesics					
IV analgesics	1.53±0.55		1.91±0.51		0.001
Oral analgesics	1.38±0.52		1.51±0.54		0.08

Blood samples were collected from all patients before surgery and again 48 hours postoperatively. The blood analysis included measurements of hemoglobin, total leukocyte count, differential leukocyte count, platelet count, erythrocyte sedimentation rate (ESR), and C-reaction protein (CRP). The results were compared between the two groups, with the findings summarized in Table 3.

**Table 3:** Preoperative and postoperative changes in acute phase reactants among the ultrasonic-activated scalpel (harmonic scalpel) and monopolar groups

Parameter	Preoperative	Postoperative	Mean	p-value
Ultrasonic-ativated scalpel (harmonic scalpel)				
Hemoglobin (g/dl)	11.84±1.59	11.71±1.48	0.13±0.57	0.02
Total leucocyte count	7980.09±1841.21	8351.30±2043.43	371.21±1701.80	0.03
Neutrophil	65.46±8.39	67.82±6.84	2.36±8.28	0.005*
Lymphocyte	28.52±8.33	26.55±7.71	1.97±8.55	0.02*
Eosinophil	2.61±1.79	2.21±1.21	0.40±2.03	0.05
Monocyte	3.22±2.06	3.11±1.86	0.11±2.21	0.62
Platelet	3.70±16.90	2.04±0.62	1.66±16.94	0.32
Erythrocyte sedimentation rate	19.87±14.88	24.22±13.92	4.35±19.66	0.02
C-reaction protein	1.03±2.41	1.85±2.92	0.81±3.84	0.03
Monopolar group				
Hemoglobin (g/dl)	12.11±1.61	11.89±1.65	0.21±0.51	0.001
Total leucocyte count	7690.36±1753.07	8654.89±2579.56	964.53±2060.30	0.0001
Neutrophil	64.34±7.32	70.39±7.94	6.05±9.38	0.0001
Lymphocyte	28.78±6.59	23.40±7.33	5.38±8.26	0.0001
Eosinophil	3.06±1.91	2.32±1.53	0.74±2.37	0.002
Monocyte	3.50±2.04	3.16±2.07	0.34±2.46	0.17
Platelets	1.83±0.54	1.84±0.58	0.01±1.23	0.02
Erythrocyte sedimentation rate	15.48±9.27	34.53±17.63	19.05±17.92	0.0001*
C-reaction protein	0.50±0.81	2.47±2.76	1.97±2.84	0.0001*

Additionally, postoperative variations in acute phase reactants between the ultrasonic-activated scalpel (UAS) and monopolar groups are compared in Table 4.

**Table 4:** Comparison of postoperative parameters between ultrasonic activated scalpel (harmonic scalpel) and monopolar cautery

Parameter	Ultrasonic-activated scalpel	Monopolar cautery	p-value
Hemoglobin (g/dl)	11.71±1.48	11.89±1.65	0.41
Total leucocyte count	8351.30±2043.43	8654.89±2579.56	0.35
Neutrophil	67.82±6.84	70.39±7.94	0.01
Lymphocyte	26.55±7.71	23.40±7.33	0.003
Eosinophil	2.21±1.21	2.32±1.53	0.57
Monocyte	3.11±1.86	3.16±2.07	0.85
Erythrocyte sedimentation rate	24.22±13.92	34.53±17.63	0.0001*
C-reactive protein	1.85±2.92	2.47±2.76	0.0001*

Ultrasonographic changes in the two groups are compared and detailed in Table 5.

**Table 5:** Comparison of ultrasonography findings between ultrasonic-activated scalpel (harmonic scalpel) and monopolar cautery

Variables	Ultrasonic-activated scalpel (harmonic scalpel) (n=100)		Monopolar ccautery (n=100)	
	N	%	N	%
Before surgery				
Symptomatic	92	92	88	88
Asymptomatic	8	8	12	12
Postoperative USG findings				
Symptomatic	0	0	0	0
Asymptomatic	0	0	0	0

## Discussion

Postoperative pain in the case of gallbladder surgeries depends on the duration of surgery and the bile leak. In a study conducted by Kannan et al., they mentioned that a harmonic scalpel is associated with less incidence of postoperative pain [13]. The monopolar cautery is associated with more postoperative pain owing to more thermal damage as compared to the UAS group [14]. In the present study, the analgesic amount requirement in the UAS group is significantly less than in the monopolar group, especially in the first 24 hours. Additionally, there were no notable changes in temperature observed in either group.

Regarding the clinical relevance of postoperative hemoglobin testing in normal individuals, Chamsy et al., in 2014, examined its significance following total laparoscopic hysterectomy and suggested that this test should be reserved for patients showing signs or symptoms suggestive of acute anemia [15]. In the present study, the change in the hemoglobin was not significant among the two groups. This also suggests that laparoscopic surgeries are very safe procedures, and the amount of blood loss is not very significant, which can lead to any drop in hemoglobin or any need for blood transfusions. When comparing total leucocyte count as an inflammatory marker, it was observed that there was no significant change in the counts among the two groups. However, Kohli et al. in 2014 mentioned a twofold rise among patients undergoing open cholecystectomy compared to those undergoing laparoscopic cholecystectomy, thus concluding that laparoscopic cholecystectomy is less traumatic than open surgery [16]. Dionigi et al. observed a reduction in total lymphocyte count in the laparoscopic group compared to the open surgery group, leading to the conclusion that laparoscopic cholecystectomy (LC) was associated with a lower acute phase response and less trauma [6]. In our study, we found a decrease in lymphocyte count in the UAS group compared to the monopolar coagulation (MC) group, indicating that the UAS approach is less traumatic. Papaziogas et al. noted a marginal increase in platelet count following both open and laparoscopic surgeries, albeit statistically insignificant [17]. In contrast, Crema et al. reported that open surgeries induced higher and more prolonged surgical stress compared to laparoscopic procedures, yet no significant differences in platelet kinetics were observed between the two techniques [18]. Both studies underscored the heightened acute phase response associated with open surgery. In our study, we observed a decrease in platelet count postoperatively in patients treated with the harmonic scalpel, whereas an increase was noted among those treated with the monopolar device. Consequently, it can be inferred that the use of the harmonic scalpel is linked to lesser inflammatory changes compared to the monopolar group.

In 2002, Grande et al. investigated the systemic acute-phase response following laparoscopic and open cholecystectomy. They measured concentrations of interleukin-6 (IL-6), interleukin-1 (IL-1), tumor necrosis factor (TNF), and C-reactive protein (CRP) before and after the operation. Their findings showed that there was no significant difference between the two groups in IL-1 and TNF response. However, the rise in plasma IL-6 levels and CRP was more pronounced after open cholecystectomy compared to the laparoscopic procedure. They concluded that the acute-phase response was less intense following laparoscopic cholecystectomy, suggesting a reduction in tissue trauma [19]. In 2000, Demirer et al. conducted a randomized study comparing postoperative acute-phase reactants in patients undergoing laparoscopic versus open cholecystectomy. They found that acute-phase reactants and length of hospitalization were significantly lower in patients undergoing laparoscopic cholecystectomy compared to those undergoing open cholecystectomy. Their conclusion suggested that laparoscopic cholecystectomy results in a less intense stress response and less tissue damage than open cholecystectomy [20]. In the present study, erythrocyte sedimentation rate (ESR) and C-reactive protein (CRP) were evaluated. Significant increases in ESR were observed in both the harmonic and monopolar groups postoperatively. However, the increase in ESR was significantly lower in the harmonic group compared to the monopolar group. Similarly, significant increases in CRP were noted in both groups postoperatively, with the increase being significantly lower in the harmonic group compared to the monopolar group.

In 1993, Ascher et al. conducted a study on postoperative sonographic findings in laparoscopic cholecystectomy (LC). They evaluated 17 consecutive patients undergoing elective LC through transabdominal ultrasound before, one day after, and six days after the procedure to identify any changes occurring in the surgical bed and surrounding tissue. The study found that the most common postoperative finding was focal sonolucency in the hepatic parenchyma adjacent to the gallbladder fossa in 35% of patients. Additionally, 29% of patients had postoperative fluid collections in the gallbladder fossa, particularly in cases where gallbladder dissection from the liver was challenging during surgery. The study highlighted that caution should be exercised before attaching significance to isolated imaging findings, as gallbladder fossa fluid may persist for up to six days after uncomplicated LC [21].

In our observation, most patients in both the harmonic (92%) and monopolar (88%) groups were symptomatic, with only a small percentage being asymptomatic (8% in harmonic and 12% in monopolar). On the seventh postoperative day, ultrasound examinations did not reveal any abnormalities in either group.

## Conclusions

The conclusion drawn from the above findings indicates that laparoscopic cholecystectomy performed with a harmonic scalpel is associated with a reduced tissue response and less tissue damage compared to the monopolar group. Consequently, the morbidity associated with the harmonic scalpel is lower than that of the monopolar group. Hence, laparoscopic cholecystectomy utilizing a harmonic scalpel is deemed a safe procedure.
